# Trauma Matters: Integrating Genetic and Environmental Components of PTSD

**DOI:** 10.1002/ggn2.202200017

**Published:** 2022-09-07

**Authors:** Shelby Marchese, Laura M. Huckins

**Affiliations:** ^1^ Pamela Sklar Division of Psychiatric Genomics Icahn School of Medicine at Mount Sinai New York NY 10029 USA; ^2^ Department of Genetics and Genomic Sciences Icahn School of Medicine at Mount Sinai New York NY 10029 USA; ^3^ Department of Psychiatry Icahn School of Medicine at Mount Sinai New York NY 10029 USA; ^4^ Seaver Autism Center for Research and Treatment Icahn School of Medicine at Mount Sinai New York NY 10029 USA; ^5^ Present address: Department of Psychiatry Yale University School of Medicine New Haven CT 06511 USA

**Keywords:** genetics, posttraumatic stress disorder, stress, trauma

## Abstract

Trauma is ubiquitous, but only a subset of those who experience trauma will develop posttraumatic stress disorder (PTSD). In this review, it is argued that to determine who is at risk of developing PTSD, it is critical to examine the genetic etiology of the disorder and individual trauma profiles of those who are susceptible. First, the state of current PTSD genetic research is described, with a particular focus on studies that present evidence for trauma type specificity, or for differential genetic etiology according to gender or race. Next, approaches that leverage non‐traditional phenotyping approaches are reviewed to identify PTSD‐associated variants and biology, and the relative advantages and limitations inherent in these studies are reflected on. Finally, it is discussed how trauma might influence the heritability of PTSD, through type, risk factors, genetics, and associations with PTSD symptomology.

## Graphical abstract

This review analyzes and explores past and current studies of posttraumatic stress disorder (PTSD) genetics and the integration of traumatic exposure. The authors recommend several methods of trauma quantification to increase genetic discovery power and improve the field's understanding of the genetic etiology of PTSD, which are critical to the discovery of novel biomarkers and precision drug targets.

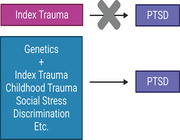

## 1 Background

Trauma exposure is inherent to the diagnosis of posttraumatic stress disorder (PTSD), but the relative importance of trauma chronicity, quantity, timing, severity, and type in relation to PTSD risk, alone and in concert with genetic factors, remains largely underexplored. An average of 65–89% of all individuals will face a significant traumatic or stressful life event (TSLE) in their lifetime (i.e., an index trauma),^[^ ^1^, ^2^, ^3^ ^]^ but only 10–30% of trauma‐exposed individuals will develop PTSD,^[^ ^4^, ^5^ ^]^ PTSD affects 6–10% Americans adults^[^ ^1^, ^6^, ^7^, ^8^ ^]^ and is associated with numerous health^[^ ^9^, ^10^, ^11^, ^12^, ^13^, ^14^, ^15^, ^16^, ^17^, ^18^, ^19^, ^20^, ^21^ ^]^ and psychosocial problems.^[^ ^22^, ^23^ ^]^ The reduced quality of life^[^ ^24^, ^25^, ^26^, ^27^ ^]^ and increased mortality risk^[^ ^9^, ^10^, ^12^ ^]^ for individuals with PTSD reflects a significant need to understand how TSLEs affect PTSD risk and genetics.

It is well known that PTSD risk is moderated by sex,^[^ ^28^, ^29^, ^30^, ^31^, ^32^, ^33^, ^34^, ^35^ ^]^ trauma type^[^ ^24^, ^36^, ^37^, ^38^, ^39^, ^40^, ^41^ ^]^ (chronicity,^[^ ^42^, ^43^, ^44^, ^45^, ^46^, ^47^ ^]^ quantity,^[^ ^48^ ^]^ developmental timing,^[^ ^42^, ^49^, ^50^, ^51^, ^52^, ^53^, ^54^ ^]^ severity^[^ ^55^, ^56^, ^57^ ^]^), race/ethnicity,^[^ ^58^, ^59^, ^60^ ^]^ personality factors,^[^ ^61^, ^62^, ^63^, ^64^, ^65^, ^66^, ^67^ ^]^ prior psychiatric disorders,^[^ ^68^, ^69^, ^70^, ^71^, ^72^ ^]^ occupation,^[^ ^73^ ^]^ other psychosocial factors,^[^ ^74^, ^75^, ^76^, ^77^ ^]^ and genetics.^[^ ^28^, ^29^, ^78^, ^79^, ^80^, ^81^ ^]^ But the specific details of how these risk factors influence PTSD risk is an active area of research. Perhaps unsurprisingly, exposure to trauma has been linked to nearly every risk factor for PTSD. Through trauma studies, the field has discovered that the risk of traumatic exposure is also influenced by sex,^[^ ^35^, ^36^, ^82^, ^83^, ^84^ ^]^ race/ethnicity,^[^ ^58^, ^59^, ^85^, ^86^, ^87^ ^]^ personality factors,^[^ ^64^, ^88^ ^]^ prior psychiatric disorders,^[^ ^89^ ^]^ occupation^[^ ^73^ ^]^ and the genetics of these risk factors. Furthermore, exposure to trauma increases an individual's risk of developing numerous psychiatric disorders, many of which are co‐morbid with PTSD (e.g., mood and anxiety disorders).^[^ ^71^, ^90^, ^91^, ^92^, ^93^, ^94^ ^]^ To disentangle the relative roles of trauma and genetics in PTSD, we need to consider not only the genetic etiology of PTSD, but also the societal and genetic factors that contribute to trauma risk.

Until the past decade, research into PTSD genetics has been limited to candidate gene and twin studies, which have been largely unsuccessful in replicating findings.^[^ ^95^ ^]^ More recently, significant advances have been made in the study of PTSD genetics through the advent of large‐scale genome‐wide association studies (GWAS).^[^ ^28^, ^29^, ^78^ ^]^ However, substantial heterogeneity among cohort definitions and collection approaches hampers progress. In particular, meta‐analyzing across cohorts of different genders and races, and with different index traumas, seems to reduce power to discover vital biomarkers and genetic associations. In order to overcome these challenges, future research must focus on understanding and quantifying trauma; in this review, we argue that analyses should move beyond case‐control to instead focus on relative resilience and vulnerability, considering whole lifetimes of TSLEs and potentially resilience‐conferring factors.

## 2 Current Progress in PTSD‐Trauma Genetics Research

### 2.1 Candidate Gene‐by‐Environment (G×E) Studies of PTSD

Prior to the scientific and technological advances that provided widespread accessibility to genome‐wide studies, candidate gene studies were the hallmark of PTSD genetics. However, candidate gene studies are limited in their discovery power due to small sample size, lack of power, and lack of adjustment for known confounders identified by GWAS.^[^ ^95^ ^]^ For this reason, the majority of PTSD candidate gene studies have failed to demonstrate replication.^[^ ^95^, ^96^ ^]^ G×E interaction findings of candidate genes without independent validation should be cautiously interpreted, as the lack of statistical power introduces a high probability of false‐positive results. Additionally, candidate gene investigations in other psychiatric disorders, such as major depressive disorder (MDD)^[^ ^97^ ^]^ and schizophrenia,^[^ ^98^ ^]^ have demonstrated a lack of reproducibility in GWAS. Together these results indicate that for psychiatric and other complex trait disorders, validation of candidate genes requires studies with large sample sizes and accurate confounder adjustment. With these caveats in mind, we have constructed an overview of past candidate G×E studies in PTSD for historical accuracy (**Table** **1**), but the resulting interactions require further validation in genome‐wide, appropriately adjusted‐for studies.

**Table 1:** Studies examining specific traumas in association with PTSD

Reference (year)	Sample size (% PTSD cases)	Mean age (SD)	Sex (% male)	Race	Primary trauma type	Genetic association	Direction of effect	*P*‐value
Candidate gene‐by‐environment (G×E) studies of PTSD								
Binder et al. (2008)	900 (% NR)	40.8 (13.8)	42.7%	95.2% AA; 2.2% EA;0.6% HA;0.1% A;0.9% Mixed; 1.0% Other	Child abuse	FKBP5	Positive	*P* < 0.0004
Xie et al. (2010)	2427 (14.0% LT)	38.6 (10.8)	54.4%	47.1% EA; 52.9% AA	Child adversity	FKBP5	Positive in AA	*P* = 0.0125
Klengel et al. (2012)	1963 (26.4% LT)	NR	NR	100% AA	Child abuse	FKBP5	Positive	*P* = 0.034
Comasco et al. (2015)	909 (% NR); 398 (% NR)	12 (0.3); 17.2 (0.68)	50.1%; 54.3%	100% Swedish ancestry	Adverse life events	FKBP5	Positive	*P* = 0.019
Watkins et al. (2016)	1585 (6.2% LT)	62.9 (14.3)	92.5%	100% EA	Child abuse	FKBP5	Positive	*P* < 0.006
Xie et al. (2009)	1252 (18.3% LT)	38.9( 11)	52.0%	46.5% EA; 53.5% AA	Adult traumatic events and child adversity	SLC6A4	Positive	*P* < 0.001
Kolassa et al. (2010)	408 (81.1% LT)	34.68 (5.9)	53.4%	100% Rwandan refugees	Rwandan genocide	SLC6A4	Positive	*P* = 0.11
Kilpatrick et al. (2007)	589 (3.2% CT)	22.6% ≤ 59; 76.6% ≥ 60	36.5%	90% EA; 3.9% AA; 3.9% HA; 1.7% Other; 0.5% Missing	2004 FL hurricanes and social support	SLC6A4	Positive (3‐way G×E)	*P* < 0.03
Grabe et al. (2009)	1663 (4.03% LT)	53.8 (14.5)	NR	100% EA	Adverse life events	SLC6A4	Positive (≥3 traumas)	*P* < 0.05
Koenen et al. (2009)	590 (3.2% CT)	<60 = 22.7%	35.1%	90.7% EA; 9.5% Other	2004 FL hurricanes	SLC6A4	Positive (unemployment/crime rate)	*P* < 0.03
Kolassa et al. (2010)	424 (80.2% LT; 48.8% CT)	34.8 (5.8)	53.4%	100% Hutu or Tutsi	Rwandan genocide	COMT	Positive	*P* = 0.04
Nelson et al. (2009)	259 (17.8% LT)	NR	NR	NR	Child abuse	GABRA2	Positive	*P* < 0.05
Amstadter et al. (2009)	607 (3.6% LT & CT)	22.6% ≤ 59; 77.4% ≥ 60	35.1%	90% EA; 3.9% AA; 3.9% HA; 1.7% Other; 0.5% Missing	2004 FL hurricanes	RGS2	Positive (low social support)	*P* < 0.001
Dunn et al. (2014)	205 (% NR)	25.8 (4.4)	3.9%	100% AA	Hurricane Katrina	RGS2	Positive (high levels of exposure)	*P* = 0.006
Lyons et al. (2013)	172 (22.7% CT)	55 (NR)	100.0%	92.3% EA; 4.2% AA; 3.5& Other	Combat exposure	APOE4	Positive	*P* < 0.014
Liberzon et al. (2014)	810 (15.1% LT); 2083 (31.5% LT)	10.4% ≥45; 40.4% ≥45	84.3%; 30.1%	100% EA; 100% AA	Child abuse	ADRB2	Positive	*P* = 1.02 × 10^−5^
Thavichachart et al. (2019)	1970 (35.6% CT)	23.1%≤ 28; 24.8% ≥49	40.0%	100% Thai ancestry	2004 Thai Tsunami	DAP1	Positive (psychosocial)	*P* < 0.001
Korem et al. (2021)	1372 (% NR)	62.1 (14.3)	91.3%	100% EA	Child abuse	CNR1	Positive	*P* < 0.001
Gene expression and genetically regulated gene expression (GReX) in PTSD								
Huckins et al. (2020)	27 538 (21.8%)	NR	93.1%	100% EA	Military	SNRNP35	Negative (prefrontal cortex)	*P* = 2.19 × 10^−7^
Marchese et al. (2022)	355 (% NR)	54.1 (8.3)	82.0%	60.8% EA; 20.3% HA; 18% AA; 2.5% NA; 1.4% A; 2.3% Other	World Trade Center disaster	CIRBP, TMSB10, FCGRT, CLIC1, RPS6KB2, HNRNPUL1, ALDOA, NACA, ZNF429, and COPE	Negative (whole blood)	*P* < 1.12 × 10^−5^
Genome‐wide association studies (GWAS) and polygenic risk score (PRS) analysis of PTSD and trauma								
Maihofer et al. (2021)	132 988 (13.3% CT & LT)	52.4 (NR)	49.7%	100% EA	Lifetime trauma exposure	SGCD, ZKSCAN2, AQP8, and STAU1	Positive/negative	*P* < 2.5 × 10^−8^
Nievergelt et al. (2015)	3494 (26.9% LT)	23.1 (3.4)	100%	85.5% EA; 4.4% AA; 10% Other	Pre‐ and post‐exposure to combat stress	PRTFDC1	Positive	*P* = 2.04 × 10^−9^
Huckins et al. (2020)	371 (% NR)	54.2 (8.4)	82.5%	60.8% EA; 20.3% HA; 18% AA; 2.5% NA; 1.4% A; 2.3% Other	WTC‐related, childhood trauma, and traumatic stress post‐9/11	PTSD PRS	Positive	*P* = 0.012
Marchese et al. (In progress)	30 468 (6.5%)	59.8 (17.9)	39.2%	34% EA; 35% HA; 25% AA; 6% Other	Lifetime traumatic or stressful life event	DPYSL2, ITGAE, PTSD PRS	Positive	*P* < 1.80 × 10^−3^
Lobo et al. (2021)	781 (% NR)	36 (13)	37%	100% EA	Motor vehicle collision	PTSD PRS	Positive (PTSD/MDD symptoms)	*P* < 0.01

NR = not reported. PTSD = posttraumatic stress disorder. EA = European American. AA = African American. HA = Hispanic American. A = Asian American.

A large majority of PTSD candidate gene studies have examined *FKBP5*, a gene involved in the hypothalamic‐pituitary‐adrenal (HPA) axis by regulating glucocorticoid receptor sensitivity. Four variants of *FKBP5* (rs9296158, rs3800373, rs1360780, and rs9470080) have been found to interact with childhood trauma through G×E interactions to significantly increase the risk of developing PTSD^[^ ^50^, ^54^, ^99^, ^100^, ^101^, ^102^, ^103^ ^]^ (Table 1). Other G×E studies in PTSD have also examined the *5HTTLPR* polymorphism (a complex‐repeat polymorphism in the 5′ upstream region of *SLC6A4*, which encodes the serotonin transporter the *5HTTLPR*) in an interaction with trauma. Significant interactions between *SLC6A4* polymorphisms and TSLEs in adulthood,^[^ ^104^ ^]^ childhood,^[^ ^104^ ^]^ exposure to genocide,^[^ ^105^ ^]^ and hurricanes^[^ ^106^ ^]^ are associated with an increased risk of developing PTSD. Other gene variants that have been studied in the context of G×E interactions for PTSD include *COMT* (Rwandan genocide),^[^ ^107^ ^]^ *GABRA2* (child abuse),^[^ ^108^ ^]^ *RGS2* (hurricanes),^[^ ^109^, ^110^ ^]^ *APOE4* (combat exposure),^[^ ^111^ ^]^ *DAP1* (tsunami and psychosocial factors),^[^ ^112^ ^]^ *ADRB2* (child abuse),^[^ ^113^ ^]^ and *CNR1* (child abuse)^[^ ^114^ ^]^ (Table 1). The majority of single nucleotide polymorphism (SNP) associations with PTSD demonstrate increased risk, but some such as variants in *RGS2*, demonstrate a differential susceptibility (i.e., some genotypes increase risk while others promote resilience).^[^ ^109^, ^110^ ^]^ While the findings of these studies suggest the presence G×E interactions in PTSD, the power of these studies is severely limited by small sample size. Independent reproduction of these candidate genes is necessary for robust, informative G×E interaction effects.

### 2.2 Genome‐Wide by Environment Interaction Studies of PTSD

As PTSD GWAS increase in sample size and variant detection power, genome‐wide by environment interaction studies (GWEIS) become more feasible. Studying G×E interactions introduces many factors that can impact significance and effect size, such as potential unknown confounding variables, exposure misclassification and dynamics, population stratification, power, and sample size.^[^ ^115^ ^]^ However, as with GWAS, the majority of these caveats can be controlled for by designing large studies with well‐defined variables, including strict, homogenous trauma exposure criteria. The majority of psychiatric GWEIS so far have been performed in MDD,^[^ ^116^, ^117^, ^118^, ^119^ ^]^ with one additional study performed on alcohol misuse,^[^ ^120^ ^]^ another in suicidality and posttraumatic stress,^[^ ^121^ ^]^ and one study in PTSD.^[^ ^87^ ^]^

Results from the GWEIS of depression indicated that SNP rs10510057 interacted with stressful life events (SLEs) and work‐related stress at nominal significance;^[^ ^119^ ^]^ rs4652467 interacted with SLEs in African Americans (AA);^[^ ^118^ ^]^ rs12789145 and rs17070072 interacted with SLEs in individuals of European ancestry;^[^ ^117^ ^]^ and rs10485715 (*BMP2*) interacted with SLEs in hospital staff.^[^ ^116^ ^]^ In alcohol misuse, *PRKG1* (variant rs1729578) significantly interacted with traumatic life experiences (TLE) in an African American cohort, and independently replicated in a secondary cohort of both African and European Americans.^[^ ^120^ ^]^

Additionally, a recent GWEIS by Wendt et al. used a linear mixed‐model approach^[^ ^122^ ^]^ to detect interactions between PTSD and genome‐wide risk variants in suicidality.^[^ ^121^ ^]^ The GWEIS identified five genome‐wide significant variants in an interaction with suicidality and PTSD case‐control status and/or quantitative PTSD risk score (the PTSD Checklist 6‐item subset, *PCL‐6*): rs12589041, rs118118557 (female‐only), rs2367967 (male‐only), rs6854286 (male‐only), and rs72619337 (male‐only).^[^ ^121^ ^]^

The results of these few initial GWEIS studies are promising, but independent replication of identified variants will be necessary to validate the G×E interactions. Particularly, replication in cohorts of varied ethnicities, gender, and traumatic experiences will be vital to understand the role these identified variants may play in PTSD and other psychiatric disorders.

## 3 Trauma Is a Strong Predictor of PTSD

### 3.1 Accounting for Trauma Increases Variance Explained

Incorporating data about cohort‐specific traumas into PTSD association models has demonstrated significant improvements in the amount of variance explained.^[^ ^87^, ^123^, ^124^ ^]^ Additionally, studies including more homogeneous cohorts have identified genome‐wide significant loci associated with PTSD, albeit without independent replication. For example, in a GWAS study with only 324 full PTSD cases of 3494 combat‐exposed male U.S. Marines and Sailors from the Marine Resiliency Study (MRS), authors were able to identify a genome‐wide significant SNP: rs6482463, mapping to *PRTFDC1* (*p* = 2.04 × 10^−9^)^[^ ^123^ ^]^ (Table 1). However, future replication of this variant is warranted to assess robustness and validity. Including other trauma exposures into the model (childhood, adult, or previous combat trauma) increased the variance explained for PTSD from ≈4% to ≈20%.

Similarly, polygenic risk score analysis using a highly homogenous cohort of 355 World Trade Center (WTC) first responders explained ≈6.5% of variance in lifetime CAPS (the Clinician Administered PTSD Scale, a quantitative measure of PTSD),^[^ ^124^ ^]^ approximately in line with polygenic risk score (PRS) estimates for other psychiatric traits (e.g., schizophrenia‐PRS^[^ ^125^ ^]^). But together, polygenic risk and exposures to traumatic stress (WTC‐related stressors, childhood trauma, and exposure to traumatic stress following 9/11) explained ≈45% of variance in lifetime CAPS and ≈48% variance in past‐month (current) CAPS. Importantly, even despite the specific and severe trauma experienced on 9/11, exposure to childhood trauma had a more significant effect on PTSD severity and chronicity in adulthood^[^ ^124^ ^]^ (Table 1).

In a multivariate GWAS analysis of trauma and PTSD: Maihofer et al.^[^ ^48^ ^]^ performed a fixed‐effects meta‐analysis GWAS on quantitative PTSD symptoms in the PGC‐PTSD Freeze 2 dataset^[^ ^29^ ^]^ (*N* = 182 199; EA), a separate GWAS of lifetime trauma exposure (LTE) burden in the UK biobank (UKBB) cohort^[^ ^126^ ^]^ (*N* = 132 988; EA), and a combined Multi‐trait analysis of genome‐wide association summary statistics (MTAG)^[^ ^127^ ^]^ multivariate analysis of both GWAS^[^ ^48^ ^]^ (Table 1). Replication analysis was performed in the latest MVP GWAS of re‐experiencing symptoms.^[^ ^78^ ^]^ The quantitative PGC‐PTSD GWAS meta‐analysis identified 5 genome‐wide significant loci, mapping to genes *GABBR1, MPP6, DFNA5, FOXP2*, and *FAM120A*. In the UKBB LTE GWAS, 6 genome‐wide significant loci were identified, mapping to *PRUNE, SGCD, FOXP2, MDGA, AC068490.2*, and *CCDC8*. The multivariate analysis of PTSD and trauma exposure revealed 4 more genome‐wide significant loci not previously identified in the meta‐analysis: *SGCD, ZKSCAN2, AQP8*, and *STAU1*.^[^ ^48^ ^]^ Of the 9 significant loci identified (5 from the PGC GWAS and 4 from the MTAG), 4 replicated significantly in the MVP cohort: rs10266297, rs10821140, rs4557006, and rs1504930 (*p* < 0.006).^[^ ^48^ ^]^ This study is the first to effectively demonstrate that incorporating trauma‐specific data can increase the effective sample size and identify more genetic risk variants than a standard GWAS in PTSD.

Last, we would like to discuss the possibility that due to the discovered genetic overlap between PTSD and trauma,^[^ ^48^ ^]^ accounting for trauma may reduce investigative power in PTSD genetic studies. However, because PTSD is a trauma‐based disorder, it is not surprising that there is genetic overlap between PTSD and trauma genetic heritability. There may be genetic factors of PTSD that interact with, or modify, genetic factors of trauma exposure—in which case researchers will need to further parse out significant variants with epigenetic and mechanistic studies. Additionally, within‐sibship genome‐wide association analyses have demonstrated significant estimated shrinkage in phenotypes sensitive to demographic and indirect genetic effects, and may be useful in determining which variants are most associated with PTSD genetics versus trauma exposure.^[^ ^128^ ^]^

## 4 Developing Strategies to Classify and Quantify Trauma in PTSD Analyses

Efforts to amass large cohorts of PTSD cases and controls must continue in order to achieve the power necessary for biomarker discovery. In order to maximize biological insights and discovery power, researchers must seek to minimize heterogeneity among both cases and controls. Ideally, cohorts should contain controls with identical trauma exposure to PTSD cases. In the event that trauma exposure differs between individuals (due to previous, unaccounted for trauma exposure such as childhood trauma), then these trauma types should be controlled for in the statistical model.

### 4.1 Prospective versus Retrospective Trauma Reporting

Before we can begin to quantify trauma types, it is important to understand the investigative reporting methods for traumas, particularly childhood traumas. Retrospective trauma reporting is typically done through self‐reported measures, possibly years after the traumatic experience.^[^ ^129^, ^130^, ^131^, ^132^ ^]^ Prospective trauma, on the other hand, is typically measured by parental reporting, or informant reporting based on surveys and interviews with parents or caregivers.^[^ ^129^, ^130^, ^131^, ^132^ ^]^ In the past, meta‐analysis studies tended to group these types of trauma reporting together, but recent analyses have highlighted significant differences in reporting, outcomes, and statistical correlations between these two methods of trauma reporting.^[^ ^129^, ^131^ ^]^ Each method has caveats: retrospective reporting is subject to memory bias, resulting in either under‐ or over‐reporting, while prospective reporting has a greater probability of under‐reporting, due to the potential repercussions of reporting maltreatment as a parent or caregiver (i.e., social, legal, etc.) and may be representative of only extreme cases of maltreatment.^[^ ^129^ ^]^

However, researchers should take note that prospective and retrospective trauma reporting are in high discordance, even between individuals. A systematic review and meta‐analysis of 16 studies (*N* = 25 471) discovered that 52% of individuals with prospective reporting did not retrospectively report, and 56% of individuals who retrospectively reported had no prospective trauma reports.^[^ ^129^ ^]^ As for which is the more accurate reporting method, more analyses of these measures are warranted. Retrospective self‐reporting is generally more accessible, but researchers should understand the limitations before combining the two reporting methods in a singular study.^[^ ^133^ ^]^ In a recent analysis of *N* = 2054 participants for childhood maltreatment reporting, authors found stronger associations with psychosocial disadvantage in individuals who reported retrospectively (OR  =  8.25, 95% CI 4.93‐13.82) versus prospective informant maltreatment reports (OR  =  2.03, 95% CI 1.36‐3.04).^[^ ^131^ ^]^

### 4.2 Trauma‐Specific Cohorts

To maximize genetic discovery power, researchers should first leverage studies of single, well‐defined situational traumas, such as the Million Veterans Project, Marine Resiliency Study, and WTC cohorts. While these focused analyses may overlook complex trauma histories, and effects may be especially significant among first responders and military cohorts^[^ ^73^ ^]^ (or other groups working in occupations with a high trauma risk), cohorts of similar trauma histories may increase effective sample size. However, to optimize study power, trauma‐specific cohort studies should also use statistical methods to account for LTE in each individual. Personality traits such as novelty‐seeking may increase risk of trauma exposure due to an increased likelihood of engaging in a high‐trauma‐risk occupation.^[^ ^134^ ^]^ These individuals experience a relative risk of PTSD threefold higher than workers who do not experience occupational trauma.^[^ ^73^ ^]^ Accordingly, efforts should be expanded to encompass common civilian traumas, such as sexual assault, domestic violence, or motor vehicle accidents.

Several studies have examined the effects of civilian traumas on PTSD risk with success. In a cohort of European American motor vehicle collision (MVC) trauma survivors, PTSD‐PRS significantly predicted risk of posttraumatic stress (2.21%) and depressive symptoms (2.77%).^[^ ^135^ ^]^ Moreover, individuals living in non‐disadvantaged neighborhoods and with college education had 47% and 52% less risk of developing posttraumatic stress.^[^ ^135^ ^]^ Studies on Rwandan refugees have also identified significant interactions of exposure to genocidal trauma with two candidate genes for PTSD: *SLC6A4* ^[^ ^105^ ^]^ and *COMT*.^[^ ^107^ ^]^ Exposure to the 2004 Florida hurricanes, in which four hurricanes made landfall in Florida in only 6 weeks causing over 144 deaths,^[^ ^136^ ^]^ also significantly interacted with *SLC6A4*,^[^ ^106^, ^137^ ^]^ and one additional candidate gene for PTSD: *RGS2*.^[^ ^109^ ^]^ In a similar disaster cohort, authors identified an association in individuals who were exposed to hurricane Katrina—which affected New Orleans in 2005, causing over 1100 fatalities—between *RGS2* and PTSD.^[^ ^110^ ^]^ However, they found that the minor allele (G) was associated with increased posttraumatic growth in higher levels of Hurricane exposure. These results suggest that *RGS2* might have a protective effect on PTSD risk.^[^ ^110^ ^]^ The culmination of these studies represents the idea that cohort homogeneity, especially leveraging single, well‐defined traumas, may increase effective sample size and power to elucidate more of the genetic architecture of PTSD.

### 4.3 Trauma‐First Analyses

Trauma‐first analyses also offer an opportunity to study the genetic etiology of different psychiatric diseases following trauma. Cross‐disorder studies indicate substantial shared genetic etiology between PTSD and other psychiatric disorders. For example, PTSD and MDD have substantial genetic overlap (34–77%),^[^ ^28^, ^138^, ^139^, ^140^ ^]^ and schizophrenia,^[^ ^28^ ^]^ alcohol dependence,^[^ ^141^, ^142^ ^]^ substance abuse,^[^ ^142^ ^]^ and nicotine dependence^[^ ^143^ ^]^ share roughly a 30–40% genetic risk for lifetime co‐occurrence with PTSD. Bipolar disorder (BP) was also identified to have a 2.5% genetic overlap with PTSD‐PRS.^[^ ^123^, ^144^ ^]^ Other cross‐disorder studies such as Genomic‐SEM (structural equation modeling; analyzes joint genetic architecture by synthesizing genetic correlations and SNP heritabilities from summary statistics) have similarly identified genetic relationships between PTSD and other psychiatric disorders such as BP, anxiety, schizophrenia, and MDD.^[^ ^145^ ^]^ However, these investigations have been hampered by the exclusion of trauma‐specific details; in order to maximize sample size, cross‐disorder studies typically include highly heterogenous cohorts and trauma exposures. By contrast, trauma‐first GWAS should recruit participants who have experienced a specific, single trauma and assess for a range of psychiatric outcomes, allowing researchers to gain insights into relative genetic and environmental contributions of trauma to a range of psychiatric disorders.

To date, very few trauma‐first analyses have yet been conducted. One recent example is a cross‐disorder study in MDD and PTSD which analyzed genetic correlations in individuals reporting trauma (traumatic life events) versus not reporting trauma.^[^ ^146^ ^]^ Authors of this study found that genetic risk for PTSD was significantly associated with MDD individuals who reported trauma (OR = 1.04), and PTSD was significantly more genetically correlated (≈20%) with higher rates of trauma reporting versus no reported trauma in individuals with MDD.^[^ ^146^ ^]^ The data from this analysis was drawn from a prior study examining SNP‐based heritability of MDD, which reported heritability of MDD with trauma exposure (24%) as greater than MDD heritability without reported trauma exposure (12%).^[^ ^91^ ^]^

Additionally, there has been one reported trauma‐first GWAS: self‐reported childhood maltreatment^[^ ^147^ ^]^ performed in a UKBB cohort (*N* = 124 000). Authors discovered two genome‐wide significant loci associated with childhood maltreatment: rs142346759 (*p*  =  4.35  ×  10^−8^, *FOXP1*) and rs10262462 (*p*  =  3.24  ×  10^−8^, *FOXP2*).^[^ ^147^ ^]^ The phenotype for childhood maltreatment was designed on a quantitative scale (0–3) for childhood physical, sexual, and emotional abuse as derived from the Childhood Trauma Questionnaire (CTQ), or otherwise reported in the UKBB. SNP heritability of childhood maltreatment was ≈6%, and while genome‐wide SNPS did not replicate in the PGC‐PTSD cohort, childhood maltreatment‐PRS was significantly predictive in PGC‐PTSD (0.25%). In a meta‐analysis of this GWAS with four additional cohorts (*N* = 185 414), authors identified 14 independent loci significantly associated with childhood maltreatment (*p* < 5 × 10^−8^).^[^ ^133^ ^]^ Of these loci, 4 genes were identified by positional mapping, expression quantitative trait loci analysis, chromatin interaction analysis, and MAGMA gene set analysis: *FES, FOXP2, SORCS3, and SAMD5*.^[^ ^133^ ^]^ Overall, the genome‐wide significant loci were associated with mental health disorders, risk‐taking, sleep difficulties, and other adverse psychosocial factors.^[^ ^133^ ^]^

To some extent, trauma‐first GWAS approaches may be approximated using electronic health records (EHR). However, we caution that trauma is likely underreported among these types of cohorts;^[^ ^148^, ^149^, ^150^, ^151^, ^152^, ^153^ ^]^ moreover, reporting of trauma will likely disproportionately take place among individuals suffering from mental illness‐ disclosed during the course of treatment or therapy.^[^ ^154^, ^155^, ^156^, ^157^, ^158^, ^159^ ^]^ As such, ascertaining sufficient trauma‐exposed controls may be difficult using this approach. Despite these caveats, some EHR‐based trauma‐first analyses have been successful in identifying psychiatric disorder risks in experiencing domestic violence,^[^ ^160^, ^161^ ^]^ sexual assault,^[^ ^161^, ^162^ ^]^ and discrimination associated with being transgender,^[^ ^163^ ^]^ homosexual or bisexual.^[^ ^164^ ^]^ Sexual assault and domestic violence were significantly associated with PTSD, anxiety, MDD, BP, eating disorders, substance use disorders, psychotic disorders, stress symptoms, and pain; acute injuries were most associated with domestic violence.^[^ ^160^, ^161^, ^162^ ^]^ Exposure to systematic discriminations was assessed in the Veterans Health Administration medical record database, and results showed that PTSD prevalence was 1.5–1.8 times higher among transgender veterans, and 2.35 times higher among homosexual and bisexual veterans.^[^ ^163^, ^164^ ^]^ Prevalence of MDD, schizophrenia, BP, substance use disorders, and military sexual trauma was also elevated among transgender veterans.^[^ ^163^ ^]^

### 4.4 Quantification of Trauma and Resilience

We urge the development of methods to quantify multiple traumas experienced across the lifespan. Such analyses might seek to assign scores to specific traumas; perform multivariate analyses across multiple traumas, or seek to fit longitudinal and whole‐phenome models to assess the degree of trauma experienced. Studies have shown that PTSD severity scores are higher when multiple traumas are included in CAPS administration than when using only a single “index” trauma,^[^ ^41^, ^165^ ^]^ perhaps reflecting more severe PTSD symptoms among individuals experiencing a large number of traumas. However, while DSM‐IV diagnosis allowed inclusion of up to three traumas, DSM‐5 diagnoses require that a single trauma be used.^[^ ^3^ ^]^

One method to address multiple traumas in PTSD that has been used is a latent class analysis (LCA) to group participants into subgroups based on trauma exposure.^[^ ^166^, ^167^, ^168^, ^169^, ^170^, ^171^, ^172^, ^173^, ^174^, ^175^ ^]^ LCA are individual‐focused, person‐centered approaches, and identify meaningful subgroups of individuals with shared patterns of trauma exposure (type, quantity, chronicity, etc.).^[^ ^176^ ^]^ Most LCA on trauma exposure have identified a three or four‐class solution, with minimal trauma making up the majority of the sample (largest class), and high and variable traumas representing the smallest classes.^[^ ^166^ ^]^ However, trauma profiles still seem to vary between cohorts, likely due to the differences in traumas experienced by individuals between cohorts (i.e., type, chronicity, severity). For example, in an LCA of 7426 African Americans, five classes were fitted from 19 different traumatic experiences: minimal trauma (i.e., infrequent trauma exposure), physical abuse (i.e., highest levels of childhood physical abuse), violence exposure (i.e., high frequency of witnessing violence), sexual abuse (i.e., highest frequency of childhood sexual abuse), and polytrauma (i.e., highest exposure to sexual assault, domestic violence, high levels of childhood physical and sexual abuse).^[^ ^174^ ^]^ The polytrauma and sexual abuse classes reported the highest PTSD symptoms, and the minimal trauma reported the fewest.^[^ ^174^ ^]^

Likewise, in a twin/sibling analysis of trauma risk, authors classified traumatic events empirically as either “low‐risk” or “high‐risk,” based on the relative risk of PTSD associated with each traumatic event (which was based on which traumas the individuals identified as most disturbing).^[^ ^140^ ^]^ An extremely high degree of genetic overlap was observed between high‐risk trauma exposure and both PTSD and MDD (89%); moreover, 47% of the variance in low‐risk trauma exposure (i.e., combat exposure, natural disaster, life‐threatening accident, witnessed injury/killing, threatened with weapon/held captive) and 60% of the variance in high‐risk trauma exposure (i.e., rape, molestation, assault, child physical abuse, child neglect) was attributable to genetics.^[^ ^140^ ^]^

Another example of how to quantify multiple traumas is to select reported traumas with high odds ratios for the disorder in question, to capture exposure to traumatic events most associated with that disorder, as demonstrated in a GWEIS of MDD; authors constructed a binary variable of resulting traumas (OR > 2.5), 7 in total, and required that an individual be exposed to two or more to be included as a trauma‐exposed case.^[^ ^91^ ^]^ Interestingly, they discovered a significant genetic correlation (*r*_g_ = 0.24) between MDD and waist circumference in trauma‐exposed individuals, but not in individuals without trauma.^[^ ^91^ ^]^

By quantifying the amount of trauma experienced, and the expected level of PTSD, we may begin to better understand which individuals are resilient and vulnerable. Importantly, individuals defined as cases within any given study may still be resilient if they experience high levels of trauma and relatively low CAPS; similarly, controls may be considered vulnerable if they experience only low levels of traumas and stressors, and exhibit high (yet sub‐clinical) CAPS. Researchers may compare resilient and vulnerable groups directly to identify risks associated with each phenotype. Few studies have explored this definition of resiliency to date. In a trauma‐first analysis of military service members, lower norepinephrine blood levels 2 months post‐deployment were associated with fewer PTSD symptoms 3 months later.^[^ ^177^ ^]^ Additionally, in a cohort of individuals in the greater New York area during the 9/11 WTC attack, authors identified multiple risk factors corresponding to resiliency (0‐2 PTSD symptoms), mild‐moderate trauma (2+ PTSD symptoms), and probable PTSD (DSM‐IV definition).^[^ ^178^ ^]^ Resilient individuals had a significantly lower incidence of depression, income loss, chronic disease, recent life stressors, past and post‐9/11 traumatic events, smoking, or marijuana use than either mild‐moderate or probable PTSD groups.^[^ ^178^ ^]^

However, stratifying already relatively small samples into discrete groups will significantly reduce power. An alternative approach is to use approaches that can quantify complex lifetime traumas to create trauma scores, and to either residualize for these to correct for the degree of trauma, or to test for interactions between trauma scores and genotype. To some extent, these will resemble studies examining the interactions between genotype and scores on trauma scales; however, this approach models all potential traumas experienced by study participants into a singular, quantitative trauma risk score (TRS). For example, in a candidate gene study of PTSD, authors combined all adult traumatic life events and exposure to childhood severity into a single trauma score; they identified significantly increased PTSD risk associated with an interaction between their TRS and the *5‐HTTLPR* genotype of the *SLC6A4* gene.^[^ ^104^ ^]^ In two more recent, previously discussed studies: a PTSD GWAS used a constructed TRS from LTE of eight different traumas in the UKBB, including “victim of a violent crime,” “life threatening illness” “witnessing death” and “experiencing sexual assault” (*N* > 20 000). By using the TRS as a covariate, they identified 9 genome‐wide significant loci.^[^ ^48^ ^]^

## 5 Caveats to Studies of Trauma‐Type Specificity in PTSD

Although stratifying by index trauma increases power of genetic association studies, I caution against some key assumptions often made when stratifying by trauma type.

First, trauma is ubiquitous. Individuals are likely to experience multiple traumas within their lifetimes, rather than a single traumatic event to which their PTSD can obviously be attributed. Incomplete surveying of trauma exposures among cases (and controls) in such studies may obscure significant relevant information. Similarly, the assumed index trauma within any given cohort may not necessarily be the most traumatic event experienced or recalled by individuals within a cohort. For example, our recent analysis of World Trade Center (WTC) first responders showed that childhood trauma was more predictive of current and lifetime highest CAPS scores than WTC exposures factors, even though our cohort is specifically ascertained for WTC‐related PTSD.^[^ ^124^ ^]^ Additionally, individuals within military cohorts may have previous civilian trauma that puts them at a greater risk of developing PTSD, as seen in the Marine Resiliency Study.^[^ ^123^ ^]^

Second, trauma is individual. Different traumas will have different salience to different individuals‐ likely due to a constellation of factors including social and family support; biological etiology (genetic and epigenetic factors); stigma associated with the specific trauma; etc.^[^ ^24^, ^36^, ^37^, ^38^, ^39^, ^40^, ^41^ ^]^ Further, broadly‐defined traumas may encompass substantially different individual experiences, including acute versus chronic (for example, abuse), violent versus psychological harm (for example, domestic violence); life‐threatening versus relatively minor (for example, motor vehicle accidents); or may occur at substantially different developmental stages or in the context of other important life events or pre‐existing stressors. It is not necessarily appropriate to assume that all individuals within a study will be traumatized to the same degree, even if they experience the same broadly‐defined trauma. In line with this reasoning, it is also unclear how to best categorize groups of traumas to assess genetic risk in terms of type or duration (e.g., domestic violence and sexual assault together because both are personal, or domestic violence with poverty because both are chronic).

Third, trauma is personal. That is, some individuals will suffer chronic, long‐term stressors and traumas due to their marginalized status (for example, due to racism, homophobia, transphobia, sexism, structural inequalities, etc.). These traumas and stressors may present differently from the single instances of trauma that are usually considered in studies of PTSD, including for example lifelong experiences of microaggressions, structural bias and inequality, and other chronic stressors.^[^ ^59^, ^60^, ^85^, ^86^ ^]^ These experiences are highly common among such marginalized groups, and elevated particularly among individuals with multiple marginalized identities,^[^ ^58^ ^]^ yet are not included in most standard scales of TSLEs, and are unasked and largely unexamined within PTSD studies. Without inquiry and careful delineation and exploration of TSLEs arising from constant and lifelong exposure to bias and inequality, we risk missing important genetic information associated with exposure to these TSLEs. Importantly, substantial genetic evidence may already exist for these effects. Large‐scale GWAS studies have consistently shown significant differences in heritability between sexes, and across different races.^[^ ^29^ ^]^ While these have been attributed to true differing underlying genetic etiology between groups, we suggest that instead, these differences may be attributable at least in part to different experiences of trauma.

## 6 How Trauma Might Influence Heritability of PTSD

Finally, we would like to discuss how different “types” of trauma might influence the heritability of PTSD. In some cohorts, specific relations between the identified genes and trauma type likely indicate true differential heritability. For example, olfactory pathways are significantly associated with PTSD in military cohorts.^[^ ^40^ ^]^ This association is genetically relevant to trauma type (combat), because PTSD sufferers experience heightened sensitivity to threat cues, such as burning odors—which are highly related to combat trauma compared to civilian trauma (e.g., childhood trauma).^[^ ^179^ ^]^

Additionally, when we discuss trauma “type” we should think critically about chronicity, severity, and developmental timing. Childhood trauma is recognized as a critical risk factor for many psychiatric disorders,^[^ ^71^, ^147^, ^180^ ^]^ but developmental timing during exposure to childhood trauma is often neglected. Instead, traumas are generally categorized as occurring in adulthood or childhood separately, but specific age/developmental period during which the trauma occurs might significantly increase the risk of mental illness.^[^ ^93^ ^]^ A recent study found that individuals exposed to childhood trauma from ages zero to five suffered from MDD and PTSD symptoms twice as high as during later developmental stages. Additionally, exposure to other forms of trauma not typically classified as childhood trauma (i.e., witnessing a friend/family member being murdered, being attacked) during ages six to ten exhibited MDD symptoms twice as high as individuals experiencing these events during adulthood.^[^ ^49^ ^]^

Severity and chronicity of trauma may also play a role in PTSD heritability. Trauma severity is particularly hard to account for, due to wide variation in measures and the personal nature of trauma; one individual may report a specific trauma as extremely traumatic and severe, while a different individual reports minimal effects from the same trauma. However, self‐reported trauma severity has been demonstrated to significantly increase PTSD risk, more than pre‐trauma factors, and thus should be incorporated into studies when possible.^[^ ^55^ ^]^ Trauma chronicity is also difficult to assess; without proper longitudinal studies on trauma and the impact on PTSD risk, current knowledge on chronicity is limited to what we know about generally chronic traumas (i.e., combat, domestic violence, abuse).

Another factor that might influence the heritability of PTSD is that different traumas may be associated with different PTSD symptoms (i.e., re‐experiencing, dysphoric arousal, anxious arousal, avoidance, and numbing).^[^ ^37^, ^94^, ^96^, ^114^, ^181^ ^]^ In line with this, different traumas—presenting different PTSD symptoms—may be more treatment resistant, and impede therapeutic practices and treatment efficacy.^[^ ^182^ ^]^ Individuals with high trauma burden often experience pronounced dissociation, depersonalization, and derealization, which results in reduced treatment responses due to a lack of activation of the fear network—crucial to effective cognitive behavioral therapy.^[^ ^183^, ^184^, ^185^ ^]^ Additionally, with the recent accessibility of epigenome‐wide studies, researchers can now more easily explore the contribution of epigenetic mechanisms that may interact with specific traumas. Studies in DNA methylation of individuals experiencing domestic violence and sexual assault have identified four genome‐wide differentially methylated genes: *BRSK2* and *ADCYAP1* in sexual assault,^[^ ^38^ ^]^ and *BDNF* and *CLPX* in domestic violence.^[^ ^186^ ^]^ Additionally, in vitro PTSD and glucocorticoid response signatures in human induced pluripotent stem cell (hiPSC)‐derived glutamatergic neurons and live cultured peripheral blood mononuclear cells represent exciting new platforms with which to test the genetic and epigenetic mechanisms underlying PTSD.^[^ ^187^ ^]^

Last, trauma exposure has been demonstrated to be heritable. Certain personality factors are heritable (i.e., harm avoidance), and can increase/decrease an individual's risk of exposure to TSLEs and subsequent PTSD.^[^ ^63^, ^64^, ^65^ ^]^ One study estimated a 20% heritability for exposure to interpersonal traumas;^[^ ^188^, ^189^ ^]^ yet another estimated 60% heritability for similar interpersonal traumas, and 47% for low‐risk traumatic events (i.e., combat exposure, natural disaster, life‐threatening accident, witnessed injury/killing, threatened with weapon/held captive).^[^ ^140^ ^]^ Additionally, exposure to combat trauma is estimated to be 30% heritable.^[^ ^190^ ^]^ These studies indicate the possibility of genetic etiology for trauma exposure, which ultimately increases the risk of developing PTSD.

## Conflict of Interest

The authors declare no conflict of interest.
